# The Composition, Structure, and Functionalities of Prolamins from Highland Barley

**DOI:** 10.3390/molecules28145334

**Published:** 2023-07-11

**Authors:** Jinjin Xing, Zhaomin Li, Wenhui Zhang, Pengjie Wang

**Affiliations:** 1 Institute of Food Science and Technology, Tibet Academy of Agricultural and Animal Husbandry Sciences, Lhasa 850030, China; xjjtibet@163.com (J.X.); lzm02503@163.com (Z.L.); 2 Department of Nutrition and Health, China Agricultural University, Beijing 100083, China; wpj1019@cau.edu.cn

**Keywords:** prolamins, highland barley, composition, structure, functionalities

## Abstract

The composition, structure, and functionalities of prolamins from highland barley were investigated. These parameters were compared with those of the commonly applied prolamins (zein). There are more charged and hydrophilic amino acids in highland barely prolamins than zein. The molecular weight of highland barely prolamins was between 30 and 63 kDa, which was larger than that of zein (20 and 24 kDa). The main secondary structure of highland barely prolamins was β-turn helices, while α-helical structures were the main secondary structure in zein. The water holding capacity, thermal stability, emulsifying capacity, and stability of prolamins from highland barley were significantly higher than in zein, while the opposite results were observed for oil absorption capacity between the two. The diameter of fibers prepared using highland barely prolamins was almost six times that of zein, while highland barely prolamins formed ribbon structures instead of fibers. Therefore, the results provide guidance for applications of prolamins from highland barley.

## 1. Introduction

Highland barley, named Qingke, is widely grown in the Qinghai–Tibet Plateau in western China [1]. Generally, about 98% of highland barley is used as feed or processed into alcoholic products [2]. Highland barley has a high starch content (more than 90% on a dry basis), making it suitable for wine production, and thus a large amount of wine lees are produced in the Qinghai–Tibet Plateau. Currently, the main use of wine lees is within animal feeds and crop fertilizers, and for the cultivation of edible fungi [3]. The mass fraction of protein in wine lees from highland barley is more than 15% (on a dry basis).

Four kinds of proteins have been established in crops, including prolamin, glutelins, albumins, and globulins [4]. Prolamin is a kind of storage protein in seeds, providing both carbon and nitrogen elements [5]. Prolamins have high content of hydrophobic amino acids (such as alanine, proline, and leucine), which can be easily dispersed in alcoholic solutions. The essential amino acid (such as lysine and tryptophan) content in prolamin is usually very low, causing a disbalance in the amino acid content of prolamins. This indicates that prolamins have little nutritional value. Prolamins can be dissolved in 60–90% alcohol solutions [6]. In recent years, they have been widely applied in the field of materials due to their unique self-assembly property, high hydrophobicity, and good biocompatibility. The unique structure and amino acid composition of prolamins render them suitable for preparing different kinds of nanoparticles, fibers, and emulsifying agents [4,7]. Thus, obtaining more information on the composition and structure of prolamins from highland barley allows for exploring further possible applications of this protein.

Based on previous reports, prolamins can be divided into various types based on their the ability to form disulfide bonds or their solubility [8]. For example, zein can be divided into α-, β-, γ-, and δ-zein, where α-zein, accounting for more than 70% of total zein, is characterized by high hydrophobicity. Similarly, hydrophobic amino acids are abundant in α-kafirin, which accounts for more than 60% of total kafirin [8]. Prolamins from different cereal seeds have also been observed to have different structures and properties [8]. For instance, due to the difference of protein conformation and aggregation, zeins, hordeins, and gliadins exhibit different electrospinnabilities and emulsifying and film-forming properties, etc. [5,9,10,11,12,13,14]. However, little is known about the structural and physicochemical properties of prolamins from highland barley, limiting its application.

The object of this study was to investigate the composition, structure, and physicochemical properties of highland barely prolamins. Wang investigated the subunit compositions, secondary structure, surface hydrophobicity, and active and total sulfydryl groups of highland barely prolamins [15]. They found that prolamins from highland barely exhibited quite different properties from prolamins from wheat. However, more information is needed before its application, including the amino acid composition, emulsifying properties, and spinning capacity. Zein, a widely researched prolamin in corn, was used for comparison [6,14]. Zein is a plant protein isolated from corn. This water-insoluble protein is one of the best understood biomacromolecules and classified as GRAS by the US Food and Drug Administration. It is unique in its ability to form an odorless, tasteless, clear, hard, and almost invisible edible coating. The findings provide basic data of the functional properties of prolamins from highland barley, and may be beneficial for the expansion of prolamin applications in the food industry.

## 2. Results

### 2.1. Amino Acid Composition

The amino acid composition determines the structural and physico-chemical properties of proteins. The amino acid composition of prolamins from highland barley is shown in Table 1. The most abundant amino acids in prolamins from highland barley were glutamic acid (41.12 g/100 g protein), proline (15.89 g/100 g protein), tryptophan (5.62 g/100 g protein), and leucine (5.03 g/100 g protein). Surprisingly, the mass percent of glutamic acid was more than 40%, which was the highest value in cereals. It is generally accepted that cysteine plays an important role in the structure and functional properties of globular proteins. Cysteine often exists in the form of free sulfhydryl groups, thiolate ion, or is oxidized into disulfide in proteins [13]. Compared with zein, prolamins from highland barley have a higher cysteine content, which facilitate their structural stability through cysteine cross-linking. The percentage of hydrophobic and hydrophilic amino acids in highland barely prolamins was significantly lower than that in zein. The ratio of acidic-to-basic amino acids plays a decisive role in the charge of proteins, which also influences their solubility [7]. Therefore, with a larger acidic-to-basic amino acid ratio and less hydrophobic amino acids, highland barely prolamins may be characterized by a higher hydratability than zein. Prolamins from highland barley were relatively deficient in lysine (0.58 g/100 protein), but this value was much larger than that in zein (0.510 g/100 protein). 

### 2.2. SDS-PAGE Analysis

**Figure 1:**
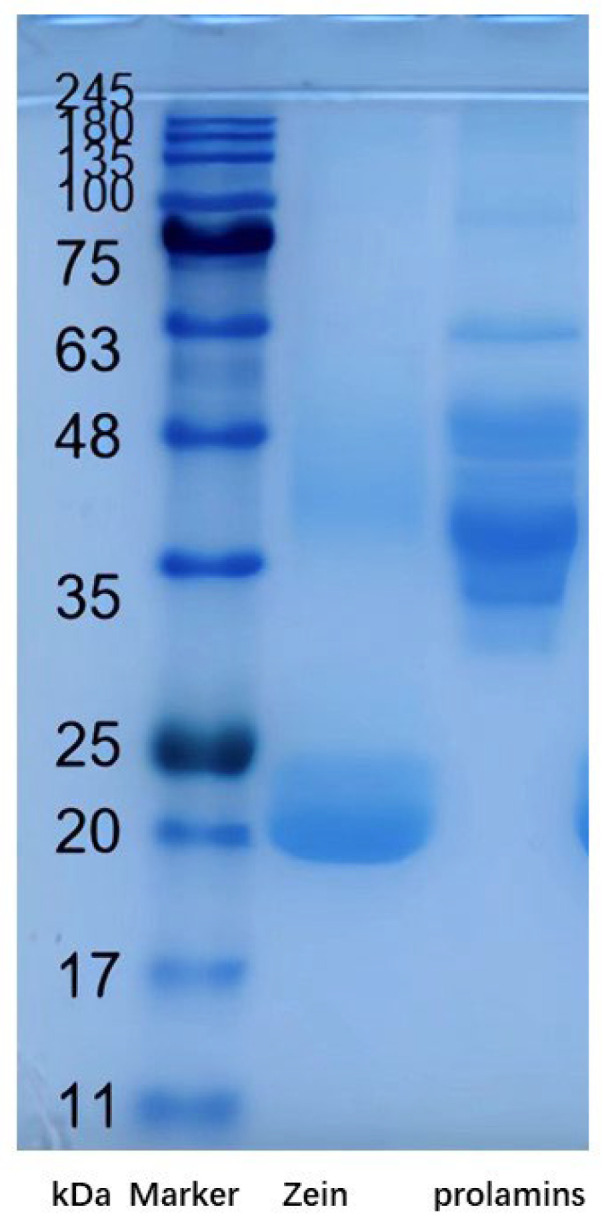
The SDS-PAGE patterns of zein and highland barely prolamins.

The SDS-PAGE profiles of prolamins from highland barley and zein are presented in Figure 1. In zein, two intense bands with molecular weights of 20 and 24 kDa were observed. A slight band at 46 kDa could also be observed in zein. This is consistent with our previous research in zein [14]. On the other hand, bands with molecular weights of 30, 45, 48, and 63 kDa in highland barely prolamins could be observed. This indicated that the molecular weights of prolamins from highland barley are significantly larger than those in zein. Almost all physico-chemical properties of proteins, including viscosity, emulsifying property, gel property, and filming-forming properties, etc., are closely related to the molecular weight [15,16]. This indicates that prolamins from highland barley may exhibit quite different functional properties from zein. 

### 2.3. Secondary Structures

**Figure 2:**
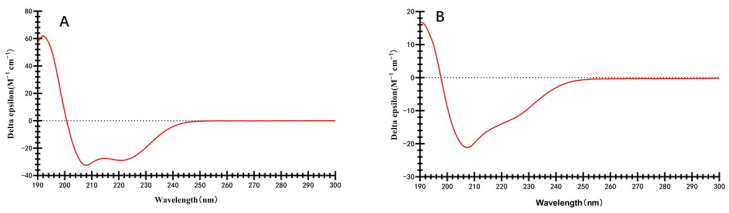
The circular dichroism spectra of zein (**A**) and highland barely prolamins (**B**).

Circular dichroism has been widely used to analyze the secondary structural characteristics of proteins [17,18]. The circular dichroism spectra of zein and highland barely prolamins are shown in Figure 2. It can be observed that prolamins from highland barley and zein exhibited quite different spectra, indicating that they have different secondary structures; although, both of them belong to the prolamin family. The circular dichroism spectra of zein have negative peaks at 222 and 208 nm, and a positive peak at about 192 nm. This indicates that the α-helical structure is the main secondary structure in zein [9]. A helical wheel structure in zein was determined, in which nine repeating subunits were in an antiparallel shape [19,20]. The circular dichroism spectra of prolamins from highland barley have a negative peak at 208 nm, while the negative peak at 222 nm disappeared. This suggested that prolamins from highland barley have less α-helical structures and more β-turn helices, which might be due to the high proportion of proline in prolamins from highland barley, inhibiting the formation of the α-helical structure. β-turn helices favor the formation of a compact and globular protein structure. Therefore, it can be speculated that prolamins from highland barley might have a more compact structure, leading to less exposure of more amino acid residues within their structure. This might endow the prolamins from highland barley more stability than zein [21,22].

### 2.4. Physico-Chemical Properties

#### 2.4.1. Thermal Properties

DSC is generally applied to characterize the thermal stability of proteins [23]. It is widely accepted that a higher peak temperature of denaturation (T_p_) indicates a higher stability of proteins. The enthalpy of denaturation (ΔH) is the energy that denatures proteins [13,24]. Table 2 shows the starting denaturation temperature (T_0_), peak temperature of denaturation (T_p_), and enthalpy of denaturation (ΔH). The T_p_ of prolamins from highland barley was 112.41 ± 2.45 °C, which is much larger than that of zein. This indicates that the thermal stability of prolamins from highland barley is higher than it is for zein. The enthalpy of denaturation of prolamins from highland barley is also larger than that of zein. After being treated, the structure of a protein changes from its native state to a denatured state, which is accompanied by the unfolding of the structure. The higher T_p_ and ΔH of prolamins from highland barley in comparison to zein may be explained through their more compact secondary structure, which is consistent with the results clarifying their secondary structures. 

#### 2.4.2. Surface Hydrophobicity

The surface hydrophobicity of proteins was evaluated with a fluorescent probe (sodium 8-anilino-1-naphthalenesulfonate), which specifically binds to the hydrophobic areas on proteins, and can thus reflect the surface hydrophobic properties of proteins [25]. It can be observed that the surface hydrophobicity index of prolamins from highland barley was lower compared to that of zein (Table 2). This indicates that the prolamins from highland barley are less hydrophobic than zein. This is largely related to the lower content of hydrophobic amino acids in highland barely prolamins, along with their less-exposed hydrophobic binding sites [13].

#### 2.4.3. Emulsifying Properties

Emulsifying properties are important processing functionalities for edible food, which refer to the protein hydrophile–lipophile balance. The emulsifying activity index refers to the ability of a protein to coat the oil–water interface, while the emulsifying stability index refers to the ability of a protein to stabilize the oil–water emulsions [26]. It was observed that the emulsifying activity index of prolamins from highland barley was significantly larger than that of zein (Table 2), indicating that the former is easier to absorb onto oil–water interfaces. The emulsifying stability index of prolamins from highland barley was also larger than that of zein, suggesting that it can stabilize oil droplets better than zein. Therefore, it was concluded that prolamins from highland barley exhibited better emulsifying properties than zein. The emulsifying properties of proteins are closely related to the hydrophile–lipophile balance. From the composition of prolamins from highland barley and zein, we observed the ratio of hydrophobic amino acids in the former were less; this could cause the water-insoluble protein to be more hydrophilic, and thus increase its ability to be adsorbed to the oil-water interface. Therefore, prolamins from highland barley and zein exhibited higher emulsifying ability and stability than zein.

### 2.5. Morphology of Electrospun Fibers

**Figure 3:**
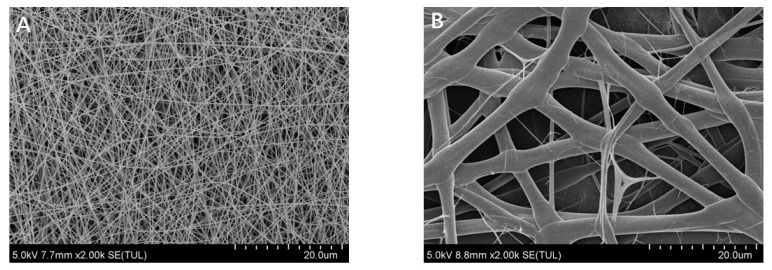
Scanning electron microscope images of fibers prepared with zein (**A**) and highland barely prolamins (**B**).

The morphology of electrospun fibers prepared with zein and highland barely prolamins are shown in Figure 3. Surprisingly, the diameter of fibers prepared with prolamins from highland barley was almost six times that of zein (2600 nm vs. 400 nm). This may be related to the higher molecular weight of prolamins from highland barley than zein, leading to a higher viscosity of highland barely prolamin dispersions than that of zein (1.042 Pa·S vs. 0.283 Pa·S) [27]. It was also observed that highland barely prolamins formed ribbon structures rather than fibers, while zein formed regular cylinder fibers. It was concluded that ribbon structures may be related to the fast evaporation of the solvent, which leads to the formation of skin in formed jets [28]. Therefore, we speculated that acetic acid evaporates slower in the presence of prolamins from highland barley than zein. This might be related to the lower ratio of hydrophilic amino acid in prolamins from highland barley, which inhibit the evaporation of solvents.

## 3. Materials and Methods

### 3.1. Materials

Zein was purchased from Sigma-Aldrich (St. Louis, MO, USA). Sodium dodecyl sulfate (SDS), 1-anilino-8-naphthalene sulfonate (ANS), dithiothreitol (DTT), hydrochloric acid (HCl), and sodium hydroxide (NaOH) were obtained from Aladdin Biochemical Technology Co., Ltd. (Shanghai, China). Ethanol was purchased from Yongda Chemical Reagent Co., Ltd. (Tianjin, China). Water was purified to 18.2 MΩ with a purifier (Sartorius Cor-poration, Göttingen, Germany). Wine lees were obtained from the local highland barley wine factory. 

### 3.2. Preparation of Prolamins from Highland Barley

Wine lees were dried to a constant weight at 70 °C in an electrothermal blowing dry box (DHG-9070B, Oulaibo, Jinan, China). The dried samples were smashed and passed through a 40 mesh sieve. 

One hundred grams of the powder were added to 1000 mL of 75% ethanol aqueous solution and mixed with a magnetic stirrer for 5 min [29,30]. Then, the samples were incubated for 120 min at 40 °C in a water bath (HH-S6, Oulaibo, Jinan, China). After, they were centrifuged for 15 min at 5000× *g* (H3-18K, Kecheng, Hunan, China) and the supernatant was poured into a 20 L stainless steel stock pot. Water (8 L) was added into the pot to precipitate the proteins. The mixture was centrifuged for 15 min at 5000× *g*, and the sediment was collected and lyophilized to a constant weight (CTFD-12S, Yonghechuangxin, Qingdao, China). 

### 3.3. Sodium Dodecyl Sulfate Polyacrylamide Gel Electrophoresis

A 12% separating gel and 4% stacking gel were used for sodium dodecyl sulfate polyacrylamide gel electrophoresis (SDS-PAGE) analyses [14]. Ten microliters of the samples (1.5 mg/mL) were loaded onto the gels and the protein markers, including 11–180 kDa, were set as a reference. Electrophoresis was performed using a Mini-PROTEAN 3 Cell electrophoresis system (Bio-Rad Laboratories, Hercules, CA, USA) at 100 V. The protein band density was analyzed using Image J software (Bethesda, MD, USA).

### 3.4. Amino Acid Composition Analyses

Twenty milligrams of the samples were added into a hydrolysis tube, along with 15 mL of 6 mol/L hydrochloric acid solution, followed by adding 3–4 drops of phenol. The hydrolysis tube was placed in the refrigerant and was frozen for 5 min. Then, the hydrolysis tube was connected to the suction pipe of the vacuum pump, vacuumed, and filled with nitrogen. After repeated vacuuming filling with nitrogen three times, the tube was sealed in a nitrogen-filled state. The sealed hydrolysis tube was hydrolyzed in an electrothermal blast incubator or hydrolysis furnace at 110 °C ± 1 °C for 22 h, and the tube was then taken out and cooled to room temperature. The hydrolysate was filtered into a 50 mL volumetric flask through an open hydrolysis tube. The hydrolysis tube was rinsed multiple times with a small amount of water, and the rinsed solution was collected in the same 50 mL volumetric flask, which was finally filled to the mark with water and shaken properly to mix the solution evenly. A precise volume of 1.0 mL of the filtrate was transferred into a 25 mL test tube. A concentrator or parallel evaporator was carried out to dry the test tube under reduced pressure at 40–50 °C. After drying, the residue was dissolved in 1–2 mL of water, and was then dried again under reduced pressure until completely dry. The dried residue was dissolved in 2.0 mL of a pH 2.2 sodium citrate buffer solution. After mixing thoroughly by shaking, the solution was filtered through a 0.22 μm filter membrane and transferred to an instrument sample vial to prepare the sample detection solution for instrument measurement. The amino acids were measured using an automatic amino acid analyzer (Hitachi L-8900, Tokyo, Japan). The amino acid composition of proteins was reported as g/100 g protein [31].

### 3.5. Circular Dichroism Spectroscopy

The protein was dispersed in a 75% ethanol solution, with a final concentration of 50 μg/mL. The Far-UV circular dichroism spectrum (190–250 nm) of the sample was measured using a circular dichroism spectrometer (MOS-450, BioLogic Inc., Digoin, France) at 25 °C [32]. 

### 3.6. Differential Scanning Calorimetry

Differential scanning calorimetry (Shimadzu, Kyoto, Japan) was applied for the thermal analysis of proteins. Briefly, 10.0 mg of the powder was added onto an aluminum plate, and was crimped. The samples were heated from 25 °C to 300 °C at 10 °C/min, and protected with nitrogen at 200 mL/min. An empty aluminum plate was used as a control [33].

### 3.7. Surface Hydrophobicity Index

The sodium 8-anilino-1-naphthalenesulfonate (10 mM) solution was prepared with a 10 mM phosphate buffer (pH 8.0), and was stored in the dark [13]. The protein dispersion, with concentrations of 1, 0.8, 0.6, 0.4, 0.2, 0.1, and 0.05 mg/mL, was prepared with 75% aqueous ethanol. During the determination, 10 μL sodium 8-anilino-1-naphthalenesulfonate solution was added to 1 mL protein dispersion, vortexed, and mixed for 30 s, and reacted for 5 min. The fluorescence intensity was measured using a multifunctional microplate detector (RF-5301PC, Shimadzu, Kyoto, Japan). The excitation wavelength was 360 nm and the emission wavelength was 460 nm. The surface hydrophobicity index of the corresponding hydrolysate was the slope obtained by plotting the concentration against the fluorescence intensity.

### 3.8. Emulsifying Properties

The sample solution, with a concentration of 0.3% (*w*/*w*), was prepared with a 10 mM phosphate buffer (pH 7.0). The solution (15 g) was put into a 50 mL centrifuge tube, and 5 g medium-chain triglyceride (MCT) was added. The sample solution was treated by a high-speed disperser at 25,000 rpm for 1 min. The sample (50 µL) was taken from the bottom of the test tube at 0 min and 10 min after stirring, respectively. The sample solution was diluted 100 times with 0.1% (*w*/*v*) SDS solution and the absorbance at 500 nm was measured. The SDS solution was used as a blank. The emulsifying activity index (EAI) and emulsifying stability index (ESI) were calculated as follows:

$$\mathrm{EAI}\;(m^{2}/g)\;=\frac{2\times 2.303\times A_{0}\times \mathrm{DF}}{c^{\times \varphi \times }\;(1\;-\;\theta )\times 10,000}$$

$$\mathrm{ESI}\;(\min)\;=\frac{A_{0}}{A_{0}\;-\;A_{10}}\times 10$$

DF is the dilution factor, DF = 100; c is protein concentration, 0.003 g/mL; φ is the optical path, φ = 0.01 m; θ is the proportion of the oil phase in the emulsion, θ = 0.25; and A_0_ and A_10_ were the absorbance at 0 min and 10 min, respectively.

### 3.9. Preparation and Characterization of the Electrospun Fibers

Dispersions containing proteins and glycerol were prepared by dissolving protein powder and glycerol into glacial acetic acid, assisted with magnetic stirring (500 rpm) at room temperature for 30 min. The final concentration of the protein and glycerol were 25% and 2.5% (*w*/*w*), respectively. The fibers were prepared on an electrospinning apparatus (HZ-11, Huizhidianfang, Qingdao, China). Electrospinning processing was performed at room temperature. The voltage was 17 kV, and the tip-to-collector distance was kept at 13 cm. The morphologies of the fibers were observed using SU8020 scanning electron microscopy (Hitachi, Tokyo, Japan).

### 3.10. Statistical Analysis

All experiments were carried out in triplicate and the results were expressed as the mean ± standard deviation. Statistical analysis used a single-factor analysis of variance (ANOVA) and Duncan’s multiple range test.

## 4. Conclusions

Prolamins from highland barley had significantly different composition, structure, and functionalities compared with the commonly used zein. With more charged and hydrophilic amino acids and a higher molecular weight, prolamins from highland barley had a higher surface charge, water holding capacity, thermal stability, emulsifying capacity and stability, and a lower oil absorption capacity. The prolamins from highland barley can be used as emulsifying agents or film-forming agents. The diameter of the fibers prepared with prolamins from highland barley was almost six times that of zein. Alongside that, highland barely prolamins formed ribbon structures rather than fibers. Therefore, these results provide guidance for applications of prolamins from highland barley.

## Figures and Tables

**Table 1:** Amino acid composition of prolamin (g/100 g protein).

Amino Acid	Zein	Prolamins from Highland Barley
Aspartic acid/Asp	5.95 ± 0.22 ^b^	2.10 ± 0.10 ^a^
Glutamic acid/Glu	26.49 ± 0.95 ^a^	41.12 ± 1.83 ^b^
Serine/Ser	6.04 ± 0.19 ^b^	3.74 ± 0.21 ^a^
Glycine/Gly	1.16 ± 0.04 ^a^	1.10 ± 0.11 ^a^
Histidine/His	1.09 ± 0.07 ^a^	1.14 ± 0.05 ^a^
Arginine/Arg	2.09 ± 0.11 ^a^	2.52 ± 0.39 ^b^
Threonine/Thr	2.16 ± 0.15 ^b^	1.80 ± 0.11 ^a^
Alanine/Ala	9.43 ± 0.31 ^b^	1.50 ± 0.10 ^a^
Proline/Pro	7.75 ± 0.30 ^a^	15.89 ± 0.87 ^b^
Tyrosine/Tyr	4.03 ± 0.02 ^b^	2.61 ± 0.09 ^a^
Valine/Val	2.38 ± 0.07 ^a^	2.13 ± 0.15 ^a^
Methionine/Met	0.11 ± 0.01 ^a^	0.11 ± 0.01 ^a^
Cystine/Cys	0.28 ± 0.02 ^b^	0.16 ± 0.06 ^a^
Isoleucine/Ile	1.89 ± 0.12 ^a^	2.02 ± 0.03 ^a^
Leucine/Leu	15.59 ± 0.34 ^b^	5.03 ± 0.16 ^a^
Tryptophan/Trp	5.12 ± 0.16 ^a^	5.62 ± 0.19 ^a^
Lysine/Lys	0.10 ± 0.01 ^a^	0.58 ± 0.04 ^b^
Hydrophobic amino acids	42.29 ± 0.59 ^b^	32.31 ± 1.05 ^a^
Hydrophilic amino acids	12.55 ± 0.77 ^b^	8.31 ± 0.45 ^a^
Acidic amino acids	32.44 ± 1.25 ^a^	43.22 ± 1.93 ^b^
Basic amino acids	3.28 ± 0.09 ^a^	4.24 ± 0.46 ^b^
Ratio of acidic-to-basic amino acids	9.87 ± 0.21 ^a^	10.24 ± 0.72 ^b^

n = 3. Different superscript letters in each line indicate statistically significant differences, *p* ≤ 0.01. Hydrophobic amino acids: Ala, Pro, Val, Met, Ile, Leu, Phe, and Trp. Hydrophilic amino acids: Ser, Thr, Cys, and Tyr. Acidic amino acids: Asp and Glu. Basic amino acids: His, Arg, and Lys.

**Table 2:** Surface hydrophobicity, surface charge, and thermal properties of zein and highland barely prolamins.

Property	Zein	Prolamins from Highland Barley
Surface hydrophobicity index	407.25 ± 35.08 ^b^	338.54 ± 21.80 ^a^
Thermal properties		
T_0_ (°C)	55.87 ± 1.40 ^a^	62.02 ± 1.85 ^b^
T_p_ (°C)	103.74 ± 3.01 ^b^	112.41 ± 2.45 ^a^
ΔH (°C)	−32.58 ± 2.40 ^b^	−55.95 ± 1.72 ^a^
Water holding capacity (g/g)	0.81 ± 0.09 ^a^	1.27 ± 0.13 ^b^
Oil absorption capacity (mL/g)	4.85 ± 0.22 ^b^	3.18 ± 0.17 ^a^
Emulsifying activity index (m^2^/g)	9.29 ± 0.81 ^a^	14.58 ± 1.05 ^b^
Emulsifying stability (min)	24.52 ± 4.19 ^a^	37.20 ± 2.41 ^b^

n = 3. Different superscript letters in each line indicate statistically significant differences, *p* ≤ 0.01.

## Data Availability

The data presented in this study are available on request from the corresponding author.
